# Identifying genetically predisposed type 1 diabetes mellitus individuals in a Southern Brazilian population: The construction of a genetic risk score

**DOI:** 10.1590/1678-4685-GMB-2023-0308

**Published:** 2025-04-18

**Authors:** Felipe Mateus Pellenz, Mayara Souza de Oliveira, Guilherme Coutinho Kullmann Duarte, Natália Emerim Lemos, Cristine Dieter, Luís Henrique Canani, Taís Silveira Assmann, Daisy Crispim

**Affiliations:** 1 Hospital de Clínicas de Porto Alegre, Serviço de Endocrinologia, Porto Alegre, RS, Brazil.; 2 Universidade Federal do Rio Grande do Sul, Faculdade de Medicina, Departamento de Clínica Médica, Programa de Pós-Graduação em Ciências Médicas: Endocrinologia, Porto Alegre, RS, Brazil.; 3 Universidade de São Paulo, Instituto de Química, Departamento de Bioquímica, São Paulo, SP, Brazil.

**Keywords:** Race adjusted-genetic risk score, HLA DR/DQ, single nucleotide polymorphisms, type 1 diabetes mellitus

## Abstract

Single nucleotide polymorphisms (SNPs) in the *HLA DR/DQ* region have the greatest impact on susceptibility to type 1 diabetes mellitus (T1DM). Non-HLA SNPs interact with the *HLA*, influencing the risk for T1DM. The aim of this study was to develop a genetic risk score (GRS) based on *HLA DR/DQ* and non-HLA SNPs to discriminate patients with T1DM. The sample comprised 466 patients with T1DM and 469 controls. The rs689/*INS*, rs2476601/*PTPN22*, rs231775/*CTLA-4*, rs2304256/*TYK2*, rs2292239/*ERBB3*, and *HLA DR/DQ* SNPs were genotyped using real-time PCR. The unweighted GRS (uGRS) was calculated by summing the risk alleles of each SNP and the weighted GRS (wGRS) by multiplying the risk alleles by their odds ratios. The uGRS was higher in T1DM patients than in non-diabetic controls (0.34 ± 0.14 *vs*. 0.26 ± 0.13, P <0.0001), being positively correlated with HbA1c levels (P <0.0001). wGRSs exhibited higher AUCs than uGRSs. The wGRS containing only *HLA DR/DQ* SNPs showed an AUC of 0.75 (95% CI 0.72 - 0.78). The wGRS containing both *HLA DR/DQ* and non-HLA SNPs, adjusted for race, demonstrated the best discriminative power [AUC 0.91 (95% CI 0.89 - 0.93)]. The race adjusted-wGRS, including all SNPs, seems to be a useful genetic tool for assessing individual’s predisposition to T1DM.

## Introduction

Type 1 diabetes mellitus (T1DM) is characterized by a severe autoimmune attack against beta-cells by macrophages and T cells, resulting in the chronic hyperglycemia, and a lifelong dependence on insulin (American Diabetes Association Professional Practice *et al.* 2022). The development of T1DM involves a complex interaction between environmental and genetic factors that modulates the communication between the immune system and beta-cells (Akil et al., 2021; American Diabetes Association Professional Practice *et al.*, 2022). The genetic component of T1DM is significant, with a concordance rate of up to 70% among monozygotic twins (Akil *et al.*, 2021). Accordingly, over 60 loci have been associated with T1DM susceptibility, with single nucleotide polymorphisms (SNPs) located in the human leukocyte antigen (*HLA*) *class II* (*DR/DQ*) region having the highest effect on T1DM susceptibility (odds ratio [OR] > 7), while non-HLA SNPs typically have ORs <2 (Winkler et al., 2014; Pociot and Lernmark, 2016; Nyaga et al., 2018; Sticht et al., 2021).

Although non-HLA SNPs have a lower individual impact on T1DM susceptibility, a study by Pociot and Lernmark (Pociot and Lernmark, 2016) showed that a genetic score including both *HLA* and non-HLA SNPs increased in 8% the screening power to detect individuals with T1DM predisposition compared to a score that only included *HLA* SNPs. This score included SNPs in *INS*, *PTPN22*, *CTLA4*, *TYK2*, and *ERBB3* genes in addition to *HLA* SNPs. Interestingly, the *INS* rs689/A and *PTPN22* rs2476601/A alleles have been linked to insulin autoantibodies presence in children with T1DM predisposition (Ilonen et al., 2013; Lempainen et al., 2015; Torn et al., 2015), while the *CTLA4* rs231775/G allele was associated with an increased risk of glutamic acid decarboxylase antibodies (GADA) (Krischer et al., 2015). The *TYK2* rs2304256/A (Pellenz et al., 2021) and *ERBB3* rs2292239/A (Lemos et al., 2018) alleles have been previously associated with T1DM in our population. B lymphoblastoid cells isolated from subjects carrying the *TYK2* rs2304256 A/A genotype demonstrated decreased STAT1 phosphorylation compared to patients with the C/C genotype, which is considered as a protective factor against T1DM development due to the downregulation of interferon-α signaling pathway. *ERBB3* is known to modulate antigen-presenting cell function and beta-cell apoptosis (Pociot and Lernmark, 2016), and children carrying the rs2292239/A allele have shown an increased risk for insulin and islet-2A autoantibodies (Maziarz et al., 2015).

With the increase in publicly available genetic data, researchers can apply various bioinformatics methods to create single variables, including SNPs in multiple genes, to facilitate the assessment and the understanding of genetic diseases (Sharp et al., 2018). The construction of genetic risk scores (GRSs) has proven to be a useful tool for predicting and classifying T1DM and T1DM-at-risk patients in large datasets (Sharp *et al.*, 2018). One of the methods to build GRSs for genetic diseases is the sum of the number of risk alleles, with further adjustments for the magnitude of effect of each locus (Abraham et al., 2013). Such methods are based on receiver operating characteristic - area under the curve (ROC AUC) analyses, which are usually used in order to assess discriminative or predictive powers, with an AUC close to 1 indicating perfect sensitivity and specificity (Hanley and McNeil, 1982).

Considering that prevention of childhood diseases such as T1DM is of medical importance and that early intervention requires tools to identify future cases, the development of T1DM-specific GRSs may set a landmark in personalized healthcare for these subjects (Winkler et al., 2012; Winkler *et al.*, 2014; Krischer et al., 2015; Bonifacio et al., 2018). Thus, the objective of this study was to construct a GRS containing SNPs in five candidate genes for T1DM (*INS*, *PTPN22*, *CTLA4*, *TYK2*, and *ERBB3*), along with SNPs in the *HLA DR/DQ* loci, in order to discriminate patients with T1DM from a Southern Brazilian population.

## Material and Methods

### Case and control samples, phenotype measurements, and laboratory analyses

This case-control study was conducted following the STROBE and STREGA guidelines (von Elm et al., 2007; Little et al., 2009). The case group included 466 patients with T1DM who were recruited from an outpatient clinic at the Hospital de Clínicas de Porto Alegre (Rio Grande do Sul, Brazil). Hyperglycemia was diagnosed according to the American Diabetes Association guidelines (American Diabetes Association Professional Practice *et al.*, 2022). In brief, subjects were diagnosed with T1DM if they were diagnosed at a young age, presented with any episode of ketoacidosis, and required insulin therapy at the time of the diagnosis. Additionally, C-peptide levels and presence of autoantibodies were measured if there were any doubts about diabetes type. The control group included 469 non-diabetic blood donors who were recruited from the same hospital. Importantly, exclusion criteria for the control group were: glycated hemoglobin (HbA1c) values ≥ 5.7% (American Diabetes Association Professional Practice *et al.*, 2022) and/or a familial history of diabetes.

Sociodemographic information (race, age, and gender) and age at T1DM diagnosis were collected from each patient from the case group and registered in a standard questionnaire. Moreover, a complete physical examination and laboratory exams were conducted in all patients, as already described by our research group (Boucas et al., 2013; Assmann et al., 2014). For control subjects, besides race, age, and gender, we also collected information on familial history of diabetes or other diseases, including type 2 diabetes mellitus, obesity, monogenic diabetes, cancer, and other chronic or genetic diseases (excepting hypertension). In addition, non-diabetic subjects had their height and weight assessed to calculate body mass index (BMI) and their serum was collected to perform HbA1c measurements. 

From all individuals in both groups, peripheral blood was collected for DNA extraction. Race was defined based on self-classification and categorized in white and non-white subjects. All subjects gave assent and written informed consent before inclusion in this study, and the Ethic Committee in Research at Hospital de Clínicas de Porto Alegre approved the study protocol (number: 2020-0226).

### Genotyping

DNA was extracted from peripheral blood using the Flexigene DNA Kit (Qiagen, MD, USA). The *TYK* rs2304256 (Pellenz et al., 2021) and the *ERBB3* rs2292239 (Lemos et al., 2018) SNPs have already been genotyped in our sample. Therefore, for this study, we specifically genotyped the *INS* rs689, *PTPN22* rs2476601, and *CTLA4* rs231775 SNPs.

The *INS* rs689(A/T) (Assay ID: C__1223317_10), *PTPN22* rs2476601(G/A) (Assay ID: C__16021387_20), and *CTLA4* rs231775(A/G) (Assay ID: C__2415786_20) SNPs were genotyped by real-time PCR technique using TaqMan SNP Genotyping Assays (Thermo Fisher Scientific, Foster City, CA, USA). Real-time PCR reactions were conducted in 384-well plates with a final volume of 5 µl, using 2 ng of genomic DNA, Mastermix TaqPath ProAmp 1× (Thermo Fischer Scientific), and TaqMan SNP Genotyping Assay 1×. Real-time PCR was performed in the ViiA7 Real-Time PCR System (Thermo Fischer Scientific) following thermal conditions suggested by the manufacturer for the Mastermix TaqPath ProAmp. Ten percent of all PCR reactions were repeated, and the genotyping success rate was >95% in our samples.

Frequencies of *HLA DR/DQ* genotypes associated with a high risk for T1DM were estimated using three SNPs (rs3104413, rs2854275, and rs9273363), as previously described (Nguyen et al. 2013). These SNPs are located in the intergenic region between *HLA-DRB1*, *HLA-DQA1*, and *HLA-DQB1*, and they are in strong linkage disequilibrium with these T1DM high-risk *HLA* genotypes, showing over 99% accuracy for their prediction (Nguyen *et al.* 2013). The rs3104413, rs2854275, and rs9273363 SNPs were previously genotyped in our samples using Custom TaqMan Genotype Assays 40 × (Thermo Fisher Scientific (Duarte et al. 2017). Following the method proposed by Nguyen *et al.* (Nguyen *et al.* 2013), we then calculated the frequencies of the following *HLA DR/DQ* genotypes: high-risk (*DR4/DQ8*or*DR3/DR4-DQ8*or*DR3/DR3*), intermediate-risk (*DR3/DRx*), and low-risk (*DRx/DRx*or*D4/DQ7*) genotypes for T1DM, where *x* can be different non-risk alleles. 

### Genetic risk score (GRS) construction and statistical analyses

Allele and genotype frequencies of the rs689/*INS*, rs2476601/*PTPN22*, and rs231775/*CTLA4* SNPs were compared between groups using χ^2^ tests. This test was also used to calculate deviations from the Hardy-Weinberg equilibrium (HWE). In addition, genotypes were compared between groups considering recessive, dominant, and additive models of inheritance (Zintzaras and Lau, 2008). These models generate an estimation of association taking into the account patterns in the inheritance of the different alleles: additive (compares aa *vs*. AA genotypes), recessive (aa+Aa *vs*. AA genotypes), and dominant (Aa+AA *vs*. aa genotypes) (Zintzaras and Lau, 2008). Unpaired Student’s *t* or χ^2^ tests were properly used to compare clinical and laboratorial characteristics between groups. Qualitative variables are shown as percentage, while quantitative variables are shown as mean ± standard deviation (SD). Moreover, univariate logistic regression analyses were used to estimate ORs with 95% confidence intervals (CI) and P values for the individual effect of rs689(T/A)/*INS*, rs2476601(G/A)/*PTPN22*, and rs231775(A/G)/*CTLA-4* SNPs (independent variables) on T1DM susceptibility (dependent variable, dichotomized into cases and controls). Moreover, multivariate analyses were performed to adjust the effect of individual SNPs for covariables (high-risk *HLA DR/DQ* genotypes and race). 

The GRS was constructed using the rs689(T/A)/*INS*, rs2476601(G/A)/*PTPN22*, rs231775(A/G)/*CTLA4*, rs2304256(C/A)/*TYK2*, rs2292239(C/A)/*ERBB3* SNPs, along with the T1DM *HLA-DR/DQ* high-risk genotypes. The unweighted GRS (uGRS) was constructed by summing the number of risk alleles of the different SNPs for each individual. The weighted GRS (wGRS) was calculated by multiplying the number of risk alleles at each locus by the estimated coefficient (OR) for the respective SNP obtained from the univariate logistic regression model. Subjects with missing data were excluded from the GRS analyses. To account for potential confounders, adjustments for race were performed using bivariate logistic regression models. ROC curve analyses were conducted to assess the diagnostic accuracy of the developed GRSs in predicting T1DM risk. The AUC was calculated for both uGRS and wGRS, considering T1DM diagnosis as a binary variable (case-control). The AUC values between the GRS models were compared using the DeLong test (DeLong et al. 1988). Moreover, aiming to perform an external validation of our data, we constructed a wGRS using ORs obtained by other studies (Oram et al., 2016; Pociot and Lernmark, 2016). This wGRS was also calculated as described above using the ORs described in the Supplementary Table S1.

Power calculation was performed at www.openepi.com. This study has a power of @ 80% (α = 0.05) to observe statistical significances for ORs < 0.65 [for the rs689/*INS* SNP (minor allele frequency - MAF = 35.0%)] or ≥ 1.50 [for the rs2476601/*PTPN22* and rs231775/*CTLA4* SNPs (MAFs of 3.0% and 43.0%, respectively)]. Statistical analyses were performed in PASW Statistics version 18.0 software (SPSS Inc., Chicago, IL, USA), and P values <0.05 were considered significant.

The Ethic Committee in Research at Hospital de Clínicas de Porto Alegre approved the study protocol (number of approval: 2020-0226). All patients and non-diabetic subjects gave assent and written informed consent prior to inclusion in the study.

## Results

### Sample description

Clinical and laboratorial characteristics of T1DM patients and non-diabetic individuals included in this study are shown in Table 1. As expected, mean HbA1c was higher in the T1DM group compared to controls. Regarding race, non-white individuals were more prevalent in the control sample. Mean age and gender distribution did not differ between groups.

**Table 1:** Characteristics of T1DM patients (cases) and non-diabetic subjects (controls).

Characteristic	Control group (n = 469)	T1DM group (n = 466)	P value^a^
Age (years)	38.3 ± 10.0	38.3 ± 12.4	0.994
Gender (% male)	56.2	51.4	0.161
T1DM duration (years)	-	20.3 ± 8.9	-
Race (% non-white)	13.6	8.9	0.030
HbA1c %	5.3 ± 0.3	8.8 ± 2.1	<0.0001
BMI (kg/m^2^)	27.1 ± 4.5	24.4 ± 3.7	<0.0001
Age at T1DM diagnosis (years)	-	17.1 ± 9.6	-

Data are shown as mean ± SD or %. BMI, body mass index; HbA1c, glycated hemoglobin; ^a^P values were obtained using χ^2^ tests or t-tests, as appropriate.

### Genotype and allele distributions of SNPs in *INS*, *PTPN22*, and *CTLA4* genes in case and control groups 

The genotype distributions of *INS* rs689, *PTPN22* rs2476601, and *CTLA-4* rs231775 SNPs were found to be in agreement with those expected by the HWE in the control group (P ≥0.050). Genotype and allele frequencies of these three SNPs in T1DM patients and controls are presented in Table 2. The frequency of the *INS* rs689 A allele was lower in the T1DM group when compared with controls (P <0.0001), and the genotype frequencies of this SNP also showed significant differences between the two groups (P <0.0001). After adjustments for T1DM high-risk *HLA DR/DQ* genotypes and race, the rs689/*INS* A/A genotype remained associated with protection against T1DM, as also observed in the additive and dominant models of inheritance.

**Table 2:** Genotype, allele frequencies of *INS* rs689, *PTPN22* rs2476601, and *CTLA-4* rs231775 SNPs, and respective odds ratios and P values in T1DM patients and non-diabetic subjects.

	Control group	T1DM group	P value^a^	Adjusted OR (95% IC) / P value^b^
***INS* rs689**	n = 466	n = 466		
**Genotypes**				
T/T	205 (44.0)	311 (66.7)	<0.0001	1
T/A	217 (46.6)	125 (26.8)		0.316 (0.223 - 0.447) / <0.0001
A/A	44 (9.4)	30 (6.5)		0.473 (0.251 - 0.893) / 0.021
**Alleles**				
T	0.673	0.801	<0.0001	-
A	0.327	0.199		
**Recessive model**				
T/T + T/A	422 (90.6)	436 (93.6)	0.115	1
A/A	44 (9.4)	30 (6.4)		0.696 (0.425 - 1.142) / 0.152
**Additive model**				
T/T	205 (82.3)	311 (91.2)	0.002	1
A/A	44 (17.7)	30 (8.8)		0.483 (0.256 - 0.910) / 0.024
**Dominant model**				
T/T	205 (44.0)	311 (66.7)	<0.0001	1
T/A + A/A	261 (56.0)	155 (33.3)		0.338 (0.243 - 0.469) / <0.0001
***PTPN22* rs2476601**	n = 469	n = 455		
**Genotypes**				
G/G	425 (90.6)	343 (75.4)	<0.0001	-
G/A	43 (9.2)	99 (21.7)		
A/A	1 (0.2)	13 (2.9)		
**Alleles**				
G	0.952	0.863	<0.0001	-
A	0.048	0.137		
**Dominant model**				
G/G	425 (90.6)	343 (75.4)	<0.001	1
G/A + A/A	44 (9.4)	112 (24.6)		2.683 (1.721 - 4.183) / <0.0001
***CTLA4* rs231775**	n = 468	n = 451		
**Genotypes**				
A/A	217 (46.4)	166 (36.8)	0.005	1
A/G	199 (42.5)	212 (47.0)		1.308 (0.935 - 1.829) / 0.117
G/G	52 (11.1)	73 (16.2)		1.533 (0.936 - 2.512) / 0.090
**Alleles**				
A	0.676	0.603	<0.001	-
G	0.324	0.397		
**Recessive model**				
A/A + A/G	416 (88.9)	378 (83.8)	0.027	1
G/G	52 (11.1)	73 (16.2)		1.579 (1.076 - 2.318) / 0.020
**Additive model**				
A/A	217 (80.7)	166 (69.5)	0.005	1
G/G	52 (19.3)	73 (30.5)		1.534 (0.933 - 2.522) / 0.091
**Dominant model**				
A/A	217 (46.4)	166 (36.8)	0.004	1
A/G + G/G	251 (53.6)	285 (63.2)		1.356 (0.988 - 1.860) / 0.059

Data are shown as number (%) or proportion. ^a^P values calculated by χ² tests. ^b^P values after adjustment for high-risk *HLA DR/DQ* genotypes and race.

The frequency of the *PTPN22* rs2476601 A allele was higher in T1DM patients compared to the control group. Similarly, the genotype frequencies of this SNP were significantly different between T1DM and control groups (P <0.0001). Under the dominant model of inheritance, the *PTPN22* rs2476601 A allele conferred an increased risk for T1DM after adjusting for T1DM high-risk *HLA DR/DQ* genotypes and race. 

The frequency of the *CTLA-4* rs231775 G allele was higher in the T1DM group when compared with controls (P= 0.001), and the genotype frequencies also differed between the two groups (P= 0.005). The G allele was initially associated with an increased risk for T1DM under the dominant model. After adjusting for T1DM high-risk *HLA DR/DQ* genotypes and race, this association was borderline.

### Genetic risk score (GRS) for T1DM prediction

MAFs of the *HLA DR4/DQ8, DR3/DR4-DQ8* or *DR3/DR* (high-risk genotypes for T1DM), *TYK2* rs230456, *ERBB3* rs2292239, *INS* rs689, *PTPN22* rs2476601, and *CTLA4* rs231775 SNPs, along with their respective ORs, used to calculate the uGRS (MAFs) and wGRS (MAFs and ORs), are provided in Table 3. These SNPs were selected based on a candidate gene approach for inclusion in the GRS models. It should be noted that data on *TYK2* rs230456 (Pellenz et al. 2021), *ERBB3* rs2292239 (Lemos et al., 2018), and the T1DM high-risk *HLA DR/DQ* (Duarte et al., 2017) SNPs in our population were previously published elsewhere. The data on the *INS* rs689, *PTPN22* rs2476601, and *CTLA4* rs231775 SNPs are reported in this study.

**Table 3:** Summary of data used to calculate the genetic risk scores.

SNP	MAF		Odds ratio (95% CI) / model	Reference
	Controls	Cases		
*INS* rs689 (T/A)	0.327	0.199	0.473 (0.251 - 0.893) / genotype	This study
*PTPN22* rs2476601 (G/A)	0.048	0.137	2.683 (1.721 - 4.183) / dominant	This study
*CTLA-4* rs231775 (A/G)	0.324	0.397	1.533 (0.936 - 2.512) / genotype	This study
*TYK2* rs2304256 (C/A)	0.296	0.231	0.467 (0.275 - 0.794) / genotype	(Pellenz et al. 2021)
*ERBB3* rs2292239 (C/A)	0.326	0.397	1.630 (1.020 - 2.610) / genotype	(Lemos et al. 2018)
High-risk *HLA DR/DQ* for T1DM*	0.175	0.571	6.415 (4.795 - 8.582) / genotype	(Duarte et al. 2017)

* *DR4/DQ8*, *DR3/DR4-DQ8* or *DR3/DR* genotypes

**Figure 1:**
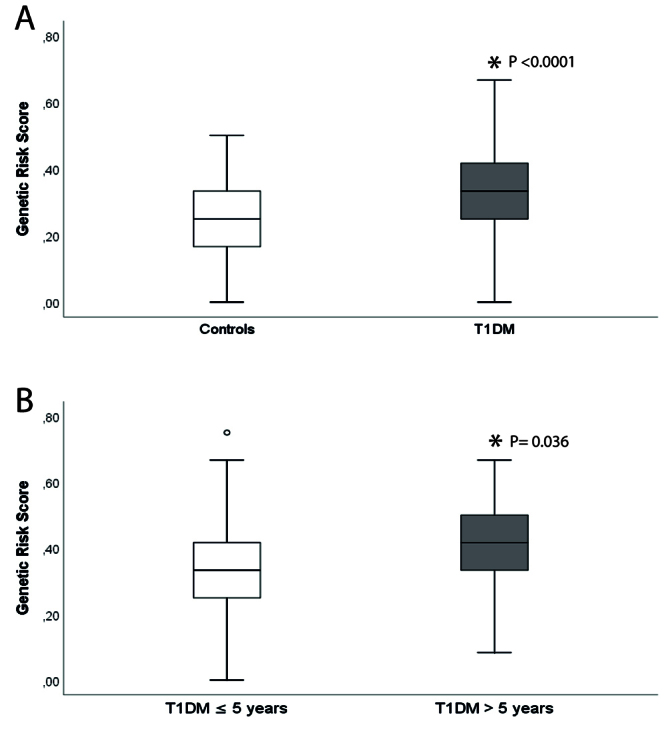
Bloxplots of the distribution of uGRS in T1DM patients and non-diabetic controls. **A)** Boxplot showing the distribution of uGRS in T1DM cases and controls. **B)** Boxplot showing the distribution of the uGRS in patients diagnosed with T1DM before the age of 5 years (T1DM ≤ 5 years) and patients diagnosed at an older age (T1DM > 5 years).

The complete uGRS was significantly higher in T1DM patients compared to controls (0.34 ± 0.14 *vs*. 0.26 ± 0.13; P <0.0001), as shown in Figure 1A. Interestingly, as depicted in Figure 1B, patients diagnosed with T1DM before the age of 5 years had a higher uGRS compared to those diagnosed at an older age (<5 years old, 0.40 ± 0.15 *vs*. > 5 years old, 0.33 ± 0.14; P= 0.036). Moreover, there was a positive correlation between HbA1c levels and the constructed uGRS (r = 0.196; P <0.0001) in both T1DM and controls.

**Figure 2:**
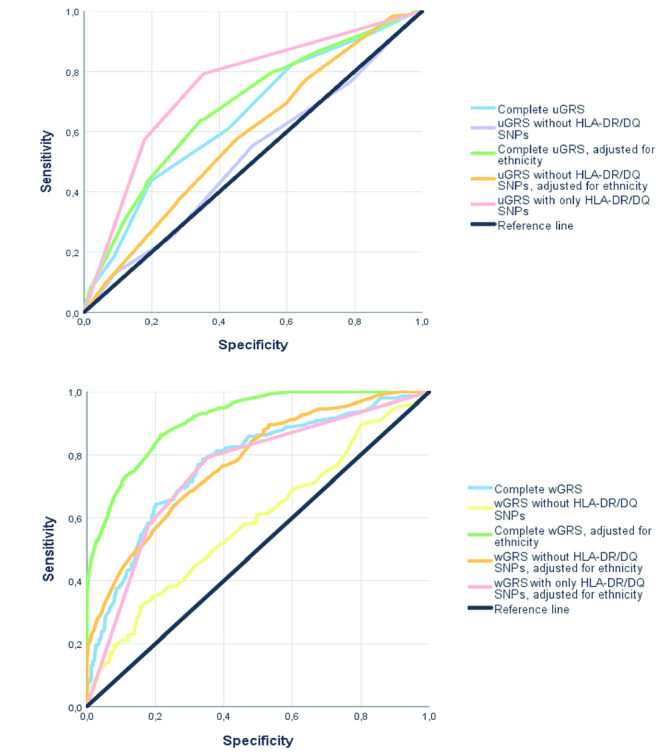
Discrimination of T1DM risk based on genetic risk scores (GRSs) comprising high-risk *HLA DR/DQ* genotypes and *TYK2* rs230456, *ERBB3* rs2292239, *INS* rs689, *PTPN22* rs2476601, and *CTLA4* rs231775 SNPs. **A)** Receiving operating characteristic (ROC) curves comparing the ability of unweighted GRSs (uGRS) to discriminate T1DM. **B)** ROC curves comparing the ability of weighted GRSs (wGRS) to discriminate T1DM.

Figure 2 and Table 4 show the ROC curves depicting the discriminatory ability of the uGRS and wGRS for T1DM. Our data demonstrate that the scores containing only high-risk *HLA DR/DQ* genotypes, without any additional non-HLA SNP, yielded a ROC AUC of 0.750 (95% CI 0.712 - 0.808) (Table 4). The uGRS containing both *HLA DR/DQ* genotypes and the 5 non-HLA SNPs yielded a ROC AUC of 0.649 (95% CI 0.608 - 0.690, unadjusted) and 0.681 (0.641 - 0.721) when adjusted for race (Figure 2A and Table 4). Notably, adjustment for race significantly improved the discriminatory ability for T1DM (P= 0.012 for the comparison with the unadjusted uGRS). 

**Table 4:** Receiver operating characteristic - area under the curve analyses to evaluate the accuracy of different genetic risk scores for discriminating T1DM.

Genetic risk score (GRS) model	AUC (95% CI)
Complete uGRS	0.649 (0.608 - 0.690)
uGRS without *HLA-DR/DQ* SNPs	0.513 (0.469 - 0.556)
Complete uGRS, adjusted for race	0.681 (0.641 - 0.721)
uGRS without *HLA-DR/DQ* SNPs, adjusted for race	0.583 (0.540 - 0.625)
Complete wGRS	0.769 (0.734 - 0.805)
wGRS without *HLA-DR/DQ* SNPs	0.587 (0.543 - 0.630)
Complete wGRS, adjusted for race	0.912 (0.892 - 0.932)
wGRS without *HLA-DR/DQ* SNPs, adjusted for race	0.774 (0.739 - 0.808)
uGRS with only *HLA-DR/DQ* SNPs	0.750 (0.712 - 0.787)
uGRS with only *HLA-DR/DQ* SNPs, adjusted for race	0.751 (0.720 - 0.783)

Data obtained by applying logistic regression analyses. AUC: area under the curve; uGRS: unweighted GRS; wGRS: weighted GRS.

The complete wGRS (*HLA DR/DQ* + non-HLA SNPs) yielded a ROC AUC of 0.769 (0.734 - 0.805), while the wGRS adjusted for race showed the highest ROC AUC [0.912 (0.892 - 0.932); Figure 2B and Table 4]. Therefore, the wGRS adjusted for race was the most accurate method in discriminating T1DM compared to the unadjusted wGRS and the adjusted uGRS (all P <0.0001). Meanwhile, the scores without *HLA DR-DQ* SNPs [uGRS: ROC AUC= 0.513 (0.469 - 0.556); wGRS: ROC AUC= 0.587 (0.543 - 0.805)] showed lower accuracies compared to the complete uGRS and wGRS (both P <0.0001 for the DeLong test), highlighting the impact of *HLA DR/DQ* genotypes in T1DM susceptibility. Interestingly, the wGRS without *HLA DR/DQ* SNPs adjusted for race showed a similar accuracy in predicting T1DM as the uGRS containing only *HLA DR/DQ* SNPs [ROC AUC = 0.774 (0.739 - 0.808) *vs*. ROC AUC = 0.750 (0.712 - 0.787), respectively; P= 0.31; Figure 2A and B, and Table 4]. Moreover, in the external validation analysis using ORs retrieved from other studies from different populations (Oram et al. 2016; Pociot and Lernmark 2016), both wGRS and race-adjusted wGRS showed similar accuracy in predicting T1DM compared to the respective calculated scores using the ORs obtained in our population, as shown in the Supplementary Table S2. Considering the impact of race on our results, we also constructed uGRS and wGRS after categorizing subjects according to race (Supplementary Table S3). In white subjects, both uGRS and wGRS were similar to the scores showed in Table 4. However, in non-white subjects, the uGRS was similar, while the wGRS was lower than the respective score showed in Table 4. However, due to the small sample size of the non-withe group (22 T1DM patients and 53 controls), this result should be interpreted with caution.

## Discussion

Considering that T1DM is a multifactorial disease triggered by several factors, including a significant impact of genetic factors on its susceptibility (Winkler et al., 2014; Pociot and Lernmark, 2016; Nyaga et al., 2018), we developed a GRS aiming to discriminating the disease in subjects from a Southern Brazilian population. Our findings demonstrated that the race-adjusted wGRS, which included *HLA-DR/DQ* genotypes and 5 non-HLA SNPs (rs689/*INS*, rs2476601/*PTPN22*, rs251775/*CTLA-4*, rs2304256/*TYK2*, and rs2292239/*ERBB3*), exhibited higher accuracy in identifying individuals at risk for T1DM compared to the other GRSs constructed in our study. To our knowledge, this is the first study that evaluated T1DM-related GRS in a Brazilian population. Thus, considering that genotype and allele frequencies of the analyzed SNPs may vary across populations, and that Brazil presents a race-mixed population, it is of upmost importance to construct a race-adjusted T1DM GRS to discriminate this disease in our population.

Thus, in this study, we genotyped rs689/*INS*, rs2476601/*PTPN22*, and rs251775/*CTLA-*4 SNPs. SNPs in the *INS* gene have the highest impact on T1DM susceptibility compared to other non-HLA SNPs (Pociot and Lernmark, 2016; Nyaga et al., 2018). Consistent with this, our findings suggest an association between the rs689/*INS* A/A genotype and protection against T1DM (OR = 0.47, 95% CI 0.25 - 0.89). The rs689 (T/A) SNP is located in an intron splice-site of the *INS* gene (Massarenti et al., 2022), and the A allele has been linked to protection against T1DM in various populations (Howson et al., 2009; Massarenti, *et al.* 2022). Furthermore, the A/A genotype of this SNP demonstrated a strong association with positivity for insulin autoantibodies (IAA) in children with T1DM from Finland (Lempainen et al. 2013). The association between the A/A genotype and increased IAA positivity can be explained by the fact that the presence of this genotype is associated with lower *INS* transcription in the thymus, leading to ineffective deletion of insulin-specific autoreactive T cells (Lempainen *et al.*, 2013; Smith et al., 2018). 

PTPN22 acts as a negative regulator of T and B cell receptors (TCR and BCR), inhibiting weak TCR/BCR ligation of activated T and B cells (Armitage et al., 2021). The rs2476601/*PTPN22* (G/A) SNP is a functional missense variant that dampens TCR/BCR signaling, thus impacting autoimmunity by increasing the number of autoreactive T and B cells that evade central tolerance (Lee and Song, 2013; Armitage *et al.*, 2021). Additionally, the rs2476601/A allele influences Treg development, as it has been linked to an increased frequency of total and näive CD4+CD25+FOXP3+ Treg cells in the peripheral blood of T1DM patients compared to controls (Valta et al. 2020). In agreement with our findings, a meta-analysis encompassing 11 studies (3,946 families) conducted in European populations showed an association between the rs2476601/A allele and an increased risk for T1DM (OR = 1.61, 95% CI 1.42 - 1.83) (Lee and Song, 2013).

CTLA-4 acts as a negative regulator of T cell activation, proliferation, and T and B cell interactions (Chen et al., 2013; Chen and Li, 2019). The rs231775/*CTLA-4* (A/G) SNP leads to the substitution of an alanine by a threonine in position 49 (Chen *et al.*, 2013). *In silico* analyses suggest that the G allele may have functional effects, as its presence reduces *CTLA-4* expression in naïve and activated Treg cells and is associated with a higher rate of IA-2A positivity (Chen *et al.*, 2018). A meta-analysis of 52 studies, including 11,017 T1DM patients and 14,191 controls, demonstrated an association between the rs231775/G allele and an increased risk for T1DM (OR = 1.41, 95% CI 1.31 - 1.53) (Chen *et al.*, 2013), which aligns with our present findings. Moreover, an updated meta-analysis of 76 studies, involving 11,420 T1DM patients and 14,674 controls, indicated an association between the G/G genotype and an increased risk for T1DM (OR = 1.27, 95% CI 1.09 - 1.50) (Chen and Li, 2019). Upon stratification analysis by race, this association remained significant in Caucasians and South Asians, but not in East Asians (Chen and Li, 2019).

Regarding the constructed GRSs, our results are consistent with the wGRS constructed by Winkler et al. (Winkler et al., 2012), which included two *HLA-DR/DQ* SNPs and 9 non-HLA SNPs, and achieved an AUC of 0.82 (0.81 - 0.83) in patients from the T1DM Genetics Consortium (T1DGC) Dataset. Their wGRS also showed high accuracy in predicting progression to T1DM or multiple islet autoantibody onset, with an AUC of 0.85 (0.84 - 0.87) (Winkler *et al.*, 2012). Another wGRS, incorporating *HLA DR/DQ* SNPs and 40 non-HLA SNPs, showed high efficacy in detecting pre-symptomatic or symptomatic T1DM patients, with an AUC of 0.68 (0.63 - 0.73) (Bonifacio et al. 2018). Additionally, Sharp et al. (Sharp et al., 2019) demonstrated that a wGRS comprising 67 non-HLA SNPs (containing the rs2476601/*PTPN22* SNP) and 18 *HLA DR-DQ* genotypes, efficiently discriminated between T1DM patients and controls from the T1DGC Dataset (AUC = 0.92; P <0.0001) (Sharp *et al.* 2019). This wGRS showed even greater discriminative ability for early-onset T1DM patients (AUC = 0.96; P <0.0001) (Sharp *et al.* 2019). Importantly, our race-adjusted wGRS achieved a similar AUC (0.912, 0.892 - 0.932) compared to other GRSs that included a higher number of SNPs (Winkler *et al.*, 2012; Sharp *et al.*, 2019).

Besides disease prediction, GRSs also demonstrate interesting applications and efficacy in discriminating different subtypes of a given disease (Patel et al., 2016; Sharp et al., 2019; Yaghootkar et al., 2019). Yaghootkar *et al* *.* (Yaghootkar *et al.*, 2019) reported that a wGRS comprising 9 SNPs (including T1DM high-risk *HLA* genotypes, rs689/*INS*, rs2476601/*PTPN22*, and rs2292239/*ERBB3* SNPs) was able to distinguish between children with monogenic diabetes or T1DM in an Iranian population, achieving an AUC of 0.90 (0.83 - 0.97) (Yaghootkar *et al.*, 2019). Similarly, Patel *et al.* (2016) applied a wGRS to discriminate between children with maturity-onset diabetes of young (MODY) or T1DM. Their score included 30 non-HLA SNPs (including rs689/*INS*, rs2476601/*PTPN22*, and rs2292239/*ERBB3*, along with T1DM high-risk *HLA* genotypes), and successfully differentiated between both diseases with an AUC of 0.87 (0.86 - 0.89) (Patel *et al.*, 2016). Moreover, the wGRS developed by Oram et al. (Oram et al. 2016), which included T1DM high-risk *HLA* genotypes and 30 non-HLA SNPs, demonstrated a strong discriminatory ability in distinguishing between T1DM and T2DM patients, with an AUC of 0.88 (0.87 - 0.89). 

Genetic susceptibility of T1DM encompasses ancestry-specific disease-associated variants (Onengut-Gumuscu et al., 2019). However, since GWASs have primarily focused on European populations, the transferability of genetic association data to non-European populations is limited (Ruan et al., 2022). Consequently, a significant limitation of GRSs is their limited generalizability across diverse ancestries and cohorts (Wang et al. 2022). In this regard, Perry et al. (Perry et al., 2018) investigated whether a wGRS, comprising T1DM high-risk *HLA DR/DQ* genotypes and 32 non-HLA SNPs, could identify T1DM patients in an ethnically diverse cohort from Southeastern U.S.A. The study showed that the constructed wGRSs successfully discriminated T1DM patients from control subjects based on their ethnicities: AUC = 0.86 for Caucasians, AUC = 0.90 for Hispanic Caucasians, and AUC = 0.75 for African-Americans (all P <0.001) (Perry *et al.,* 2018). In a study conducted among individuals of African-ancestry, a wGRS containing 5 non-HLA SNPs, along with African-specific T1DM high-risk *HLA* genotypes, showed a high discriminatory ability to predict T1DM, with an AUC of 0.87 (Onengut-Gumuscu *et al.,* 2019).

Our data should be interpreted within the context of a few limitations. First, we employed a limited set of SNPs associated with T1DM susceptibility, which implies that we cannot dismiss the possibility that other loci may exert significant effects on T1DM development. Second, allele frequencies and effect sizes for each SNP may vary across different ethnicities, potentially impeding the reproducibility of our findings in less diverse populations. Therefore, it is crucial to validate our results in other populations and ethnicities, utilizing larger sample sizes. Additionally, we did not perform internal validation of our results in other Brazilian samples; thus, further studies in our population should be conducted to validate our data. Third, we acknowledge the potential of stratification bias in our analysis; however, we mitigated this concern by including race as a covariate in the multivariate regression analyses. Fourth, in future studies, it would be interesting to include the *HLA DR15-DQ6* haplotype in the GRS, considering that it is highly protective against T1DM (Thomas et al., 2021). Fifth, subjects’ race was self-reported, and we did not evaluate the ancestry genetically; thus, this aspect may have influenced the interpretation of our results considering that ancestry shows an important impact on susceptibility to diseases, including T1DM, especially in non-Caucasian individuals (Bizzarri et al., 2015).

In conclusion, our study suggests that the race-adjusted constructed wGRS can serve as a valuable genetic tool for identifying individuals predisposed to T1DM, enabling progress in the prevention and management of this disease. Furthermore, we observed that the minor allele of the rs689/*INS* SNP was associated with protection against T1DM, whereas the minor alleles of the rs2476601/*PTPN22* and rs231775/*CTLA-4* SNPS were associated with increased risk for this disease.

## Supplementary material

The following online material is available for this article:

Table S1 - Summary of data used to calculate the genetic risk scores for external validation.

Table S2 - Receiver operating characteristic - area under the curve analyses to evaluate the accuracy of different weighted genetic risk scores for discriminating T1DM using odds ratios available in the literature.

Table S3 - Receiver operating characteristic - area under the curve analyses to evaluate the accuracy of genetic risk scores (GRS) for discriminating T1DM between white and non-white subjects.
