# Supplementary material for: Identifying genetically predisposed type 1 diabetes mellitus individuals in a Southern Brazilian population: The construction of a genetic risk score
Source: Genet Mol Biol. 2025 Apr 18;48(2):e20230308. doi: 10.1590/1678-4685-GMB-2023-0308 (PMC11999062; doi:10.1590/1678-4685-GMB-2023-0308)
Supplement: Table S1 - [file 1415-4757-GMB-48-02-e20230308-s1.pdf]

Genetics and Molecular Biology

https://doi.org/10.1590/1678-4685-GMB-2023-0308

Supplementary Material to “Identifying genetically predisposed type 1 diabetes mellitus individuals in a Southern Brazilian population: The construction of a genetic risk score”

Table S1 - Summary of data used to calculate the genetic risk scores for external validation.

SNP	Odds ratio	Reference
INS rs689 (T/A)	0.42	(Pociot and Lernmark 2016)
PTPN22 rs2476601 (G/A)	1.96	(Oram et al. 2016)
CTLA-4 rs231775 (A/G)	1.53	This study
TYK2 rs2304256 (C/A)	0.87	(Pociot and Lernmark 2016)
ERBB3 rs2292239 (C/A)	1.35	(Oram et al. 2016)
High-risk *HLA DR/DQ* for T1DM*	7.03	(Oram et al. 2016)

* *DR4/DQ8, DR3/DR4-DQ8* or *DR3/DR* genotypes.
