# Supplementary material for: Identifying genetically predisposed type 1 diabetes mellitus individuals in a Southern Brazilian population: The construction of a genetic risk score
Source: Genet Mol Biol. 2025 Apr 18;48(2):e20230308. doi: 10.1590/1678-4685-GMB-2023-0308 (PMC11999062; doi:10.1590/1678-4685-GMB-2023-0308)
Supplement: Table S2 - [file 1415-4757-GMB-48-02-e20230308-s2.pdf]

Genetics and Molecular Biology

https://doi.org/10.1590/1678-4685-GMB-2023-0308

Supplementary Material to “Identifying genetically predisposed type 1 diabetes mellitus individuals in a Southern Brazilian population: The construction of a genetic risk score”

Table S2 - Receiver operating characteristic – area under the curve analyses to evaluate the accuracy of different weighted genetic risk scores for discriminating T1DM using odds ratios available in the literature.

**Genetic risk score (GRS) model**	**AUC (95% CI)**
Complete wGRS	0.766 (0.730 – 0.802)
Complete wGRS, adjusted for race	0.912 (0.892 – 0.932)

Data obtained by applying logistic regression analyses. AUC: area under the curve; wGRS: weighted GRS.
