# Supplementary material for: Identifying genetically predisposed type 1 diabetes mellitus individuals in a Southern Brazilian population: The construction of a genetic risk score
Source: Genet Mol Biol. 2025 Apr 18;48(2):e20230308. doi: 10.1590/1678-4685-GMB-2023-0308 (PMC11999062; doi:10.1590/1678-4685-GMB-2023-0308)
Supplement: Table S3 - [file 1415-4757-GMB-48-02-e20230308-s3.pdf]

Genetics and Molecular Biology

https://doi.org/10.1590/1678-4685-GMB-2023-0308

Supplementary Material to “Identifying genetically predisposed type 1 diabetes mellitus individuals in a Southern Brazilian population: The construction of a genetic risk score”

Table S3 - Receiver operating characteristic – area under the curve analyses to evaluate the accuracy of genetic risk scores (GRS) for discriminating T1DM between white and non-white subjects.

Genetic risk score (GRS) model	Controls (N)	T1DM (N)	AUC (95% CI)
Complete uGRS (white subjects)	338	276	0.643 (0.599 – 0.687)
Complete wGRS (white subjects)	338	276	0.769 (0.731 – 0.806)
Complete uGRS (non-white subjects)	53	22	0.672 (0.538 – 0.806)
Complete wGRS (non-white subjects)	53	22	0.750 (0.631 – 0.868)

Data obtained by applying logistic regression analyses. AUC: area under the curve; uGRS: unweighted GRS; wGRS: weighted GRS.
